# Supplementary material for: Ability of prebiotic polysaccharides to activate a HIF1α-antimicrobial peptide axis determines liver injury risk in zebrafish
Source: Commun Biol. 2019 Jul 25;2:274. doi: 10.1038/s42003-019-0526-z (PMC6658494; doi:10.1038/s42003-019-0526-z)
Supplement: Supplementary file 1 — Supplementary Information [file 42003_2019_526_MOESM1_ESM.pdf]

Supplementary Information

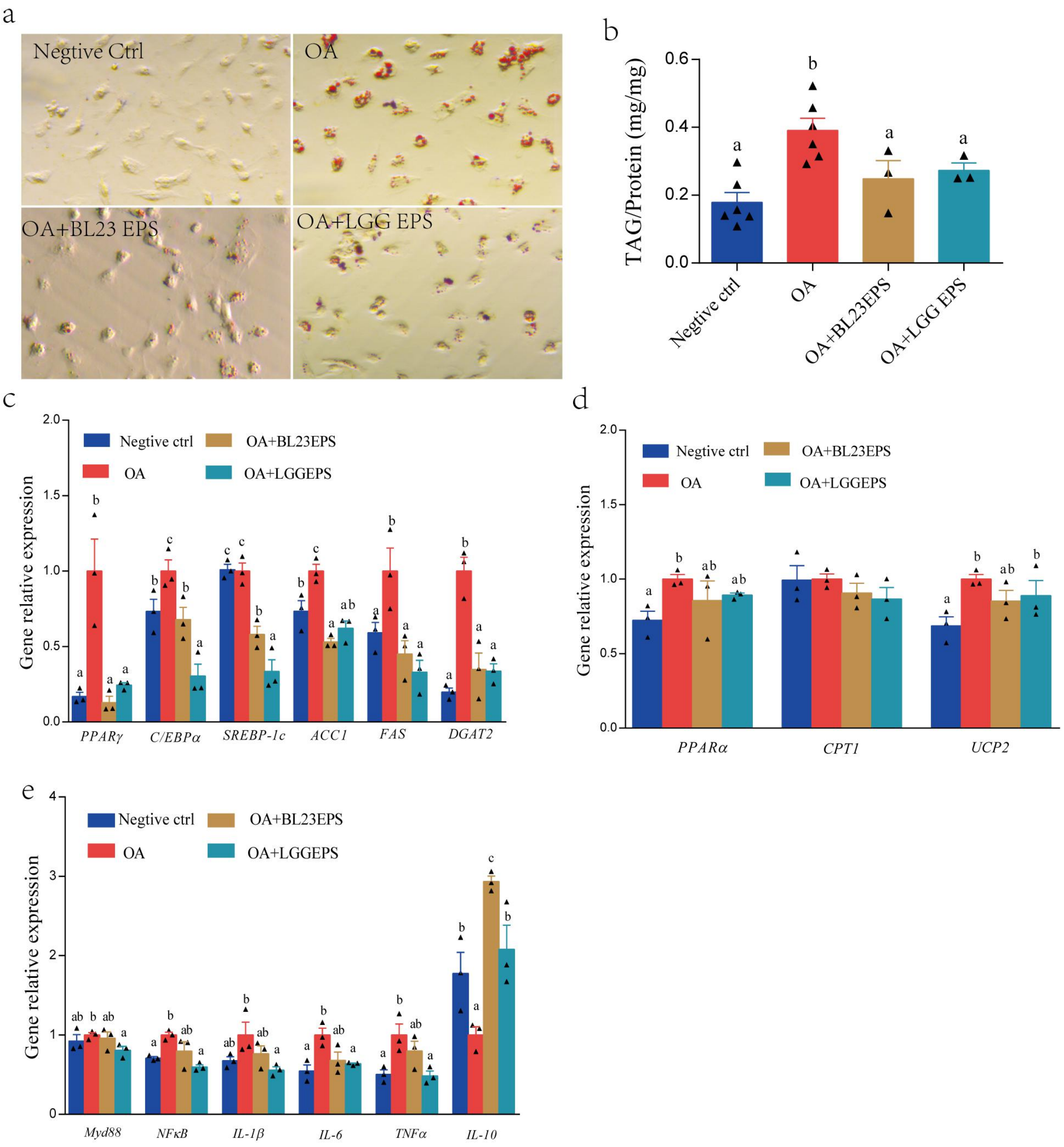

Supplementary Figure 1. LGG EPS and BL23 EPS inhibit lipogenesis and improve inflammation status in ZFL cells. ZFL cells were treated with 10.0 µg/ml LGG EPS or BL23 EPS with the addition of 100 µM oleic acid for 24h. **(a)** The profile of lipid droplet formation with Oil red O staining in ZFL. **(b)** TAG accumulation in ZFL by TAG assay (n = 3-6). The expression of genes related to lipogenesis **(c)**, energy expenditure **(d)**, and inflammation **(e)** in ZFL as measured by *q*-PCR (n = 3). Data are expressed as the mean ± SEM. Graph bars in **b–e** labelled with different letters on top represent statistically significant results ($P < 0.05$), whereas bars with the same letter correspond to results that show no statistically significant differences.

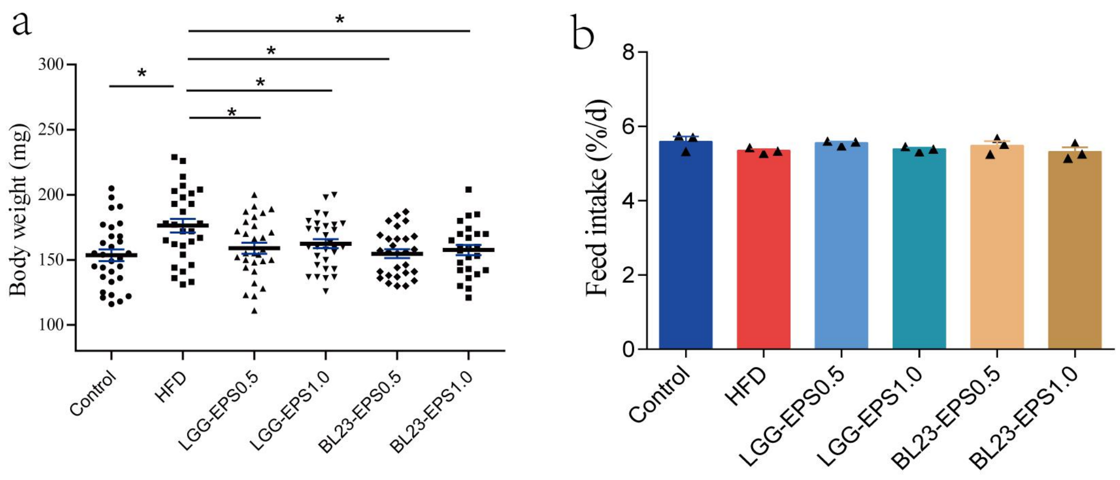

Supplementary Figure 2. The effects of EPS administration on the growth performance in HFD-fed zebrafish. Adult zebrafish (one-month-old) were fed with the control diet, HF diet, or HF diet supplemented with 0.5% or 1% EPS for four weeks. **(a)** The body weight of adult zebrafish fed on different diets for four weeks. **(b)** Food intake of adult zebrafish fed on different diets for four weeks. Data were expressed as the mean ± SEM. Differences are considered significant at $P < 0.05$ (*) and $P < 0.01$ (**).

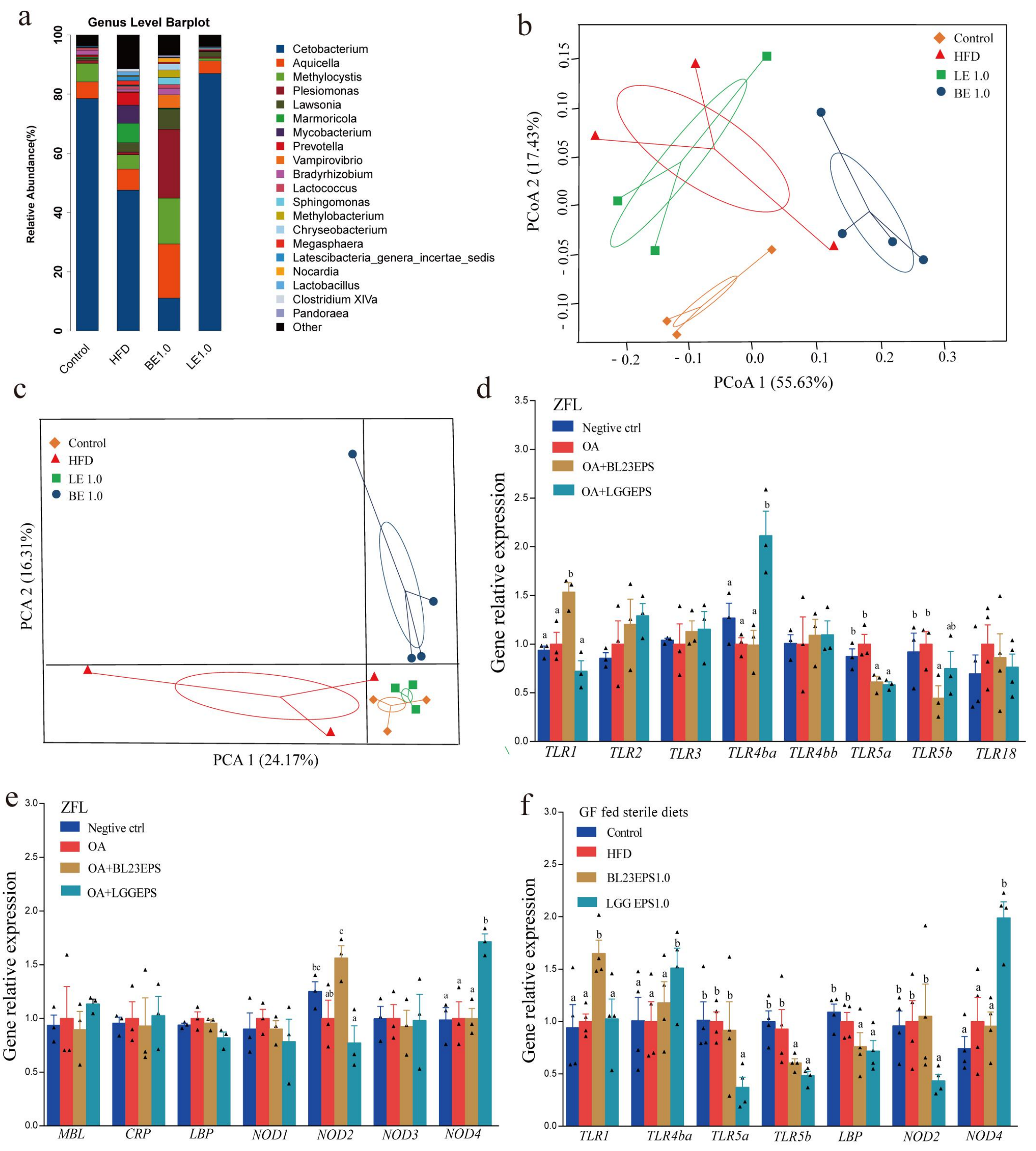

Supplementary Figure 3. The liver damage effect of BL23 EPS is mediated by the gut microbial dysbiosis. (a) The relative bacterial abundance at the level of genus of the microbiota of adult zebrafish fed with diets for four week. **(b)** The weighted version of UniFrac-based PCoA of the microbiotas of adult zebrafish fed with diets for four weeks. **(c)** Principle component analyses (PCA) of the genera with different abundance among zebrafish fed with different diets for four weeks. The expression of transmembrane PRRs (*TLRs*) genes **(d)**, secreted PRRs (*MBL, CRP, LBP*) genes, and cytosolic PRRs (*NODs*) genes **(e)** in ZFL cells treated with LGG EPS or BL23 EPS for 24h. **(f)** The expression of pattern recognition receptor (PRR) genes in GF larvae colonized with four different gut microbiotas, and fed sterile HFD diets for seven days (n = 4, pool of 20 larvae per sample). Data are expressed as the mean ± SEM. Graph bars labeled with different letters on top represent statistically significant results ($P < 0.05$), whereas bars with the same letter corresponds to results that show no statistically significant differences.

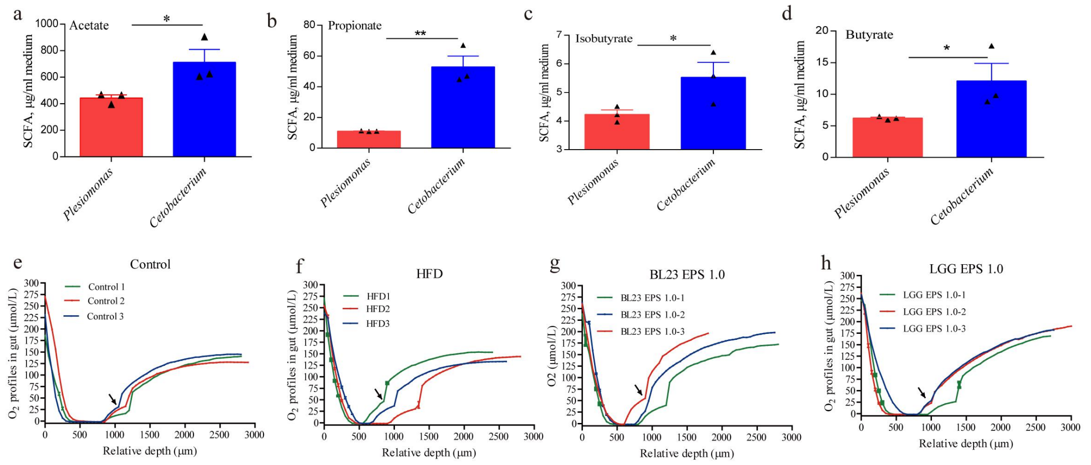

Supplementary Figure 4. Butyrate mediated the further differentiation of the LGG EPS-and BL23 EPS-associated microbiota. Acetate levels **(a)**, propionate levels **(b)**, isobutyrate levels **(c)**, butyrate levels **(d)** produced by equal CFU of *Cetobacterium* and *Plesiomonas* which were cultured in GAM (Glfu Anaerobic Medium) medium, and were incubated at 28°C for 12h under anaerobic conditions (n = 3). Profiles of O_2 concentration in gut for **(e)** Control,**(f)** HFD, **(g)** 1.0% BL23 EPS and **(h)** 1.0% LGG EPS groups. The relative depth refers the position relative to initial starting location. The points of inflection of oxygen profiles (the solid arrowheads) indicate the O_2 concentration in the intestinal mucosa layer (**Fig. 9e**) as described by Zeitouni *et al*. The figure shows the oxygen profiles of three different zebrafish midguts. Data were expressed as the mean ± SEM. Differences are considered significant at $P < 0.05$ (*) and $P < 0.01$ (**). (Zeitouni NE, Chotikatum S, von Köckritz-Blickwede M, Naim HY. (2016) The impact of hypoxia on intestinal epithelial cell functions: consequences for invasion by bacterial pathogens. Molecular and cellular pediatrics 3: 14.)

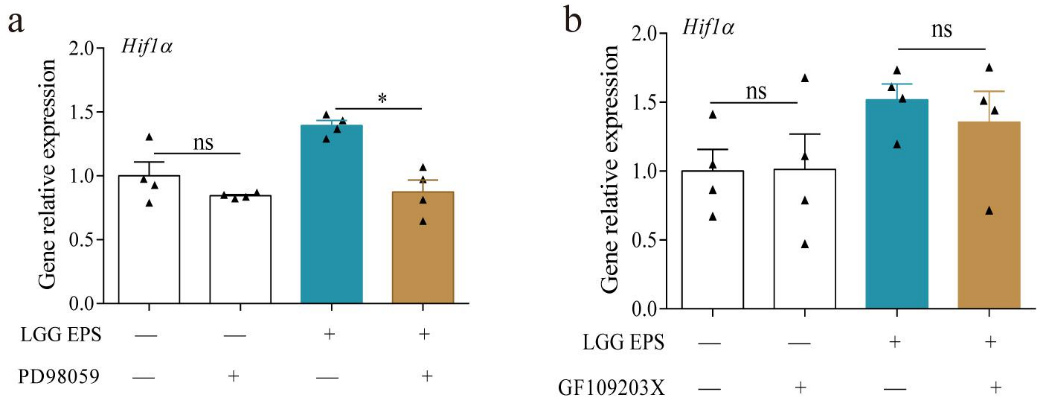

Supplementary Figure 5. Implication of the p44/42 MAPK and PKC pathways in HIF-1 induction by LGG EPS. GF zebrafish were pretreated or not for 1h with PD 98059 (50 μM) or GF109203X (10 μM) and maintained under control conditions in the presence of 10 μg/mL LGG EPS for 6 hours. The expression of *Hif-1α* **(a, b)** in the GF zebrafish treated with PD 98059 or GF109203X.

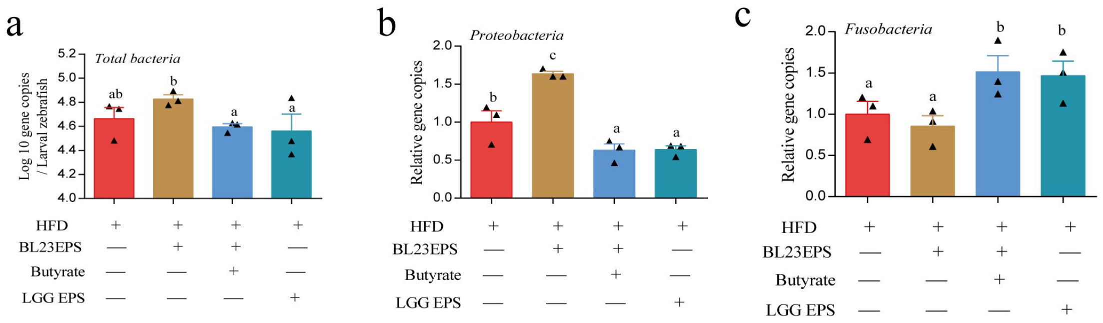

Supplementary Figure 6. The approaches to control the risks. Zebrafish larvae fed HFD, and HF diet supplemented with 1.0% BL23 EPS, 1.0% BL23 EPS plus 0.1% tributyrin, or 1.0% LGG EPS for one week. The total bacteria **(a)** and the relative bacterial abundance of Phylum *Proteobacteria* **(b)**, and Phylum *Fusobacteria* **(c)** of the microbiota of zebrafish larvae (n = 3, pool of 20 larvae per sample). Data are expressed as the mean ± SEM. Graph bars labelled with different letters on top represent statistically significant results ($P < 0.05$), whereas bars with the same letter correspond to results that show no statistically significant differences.

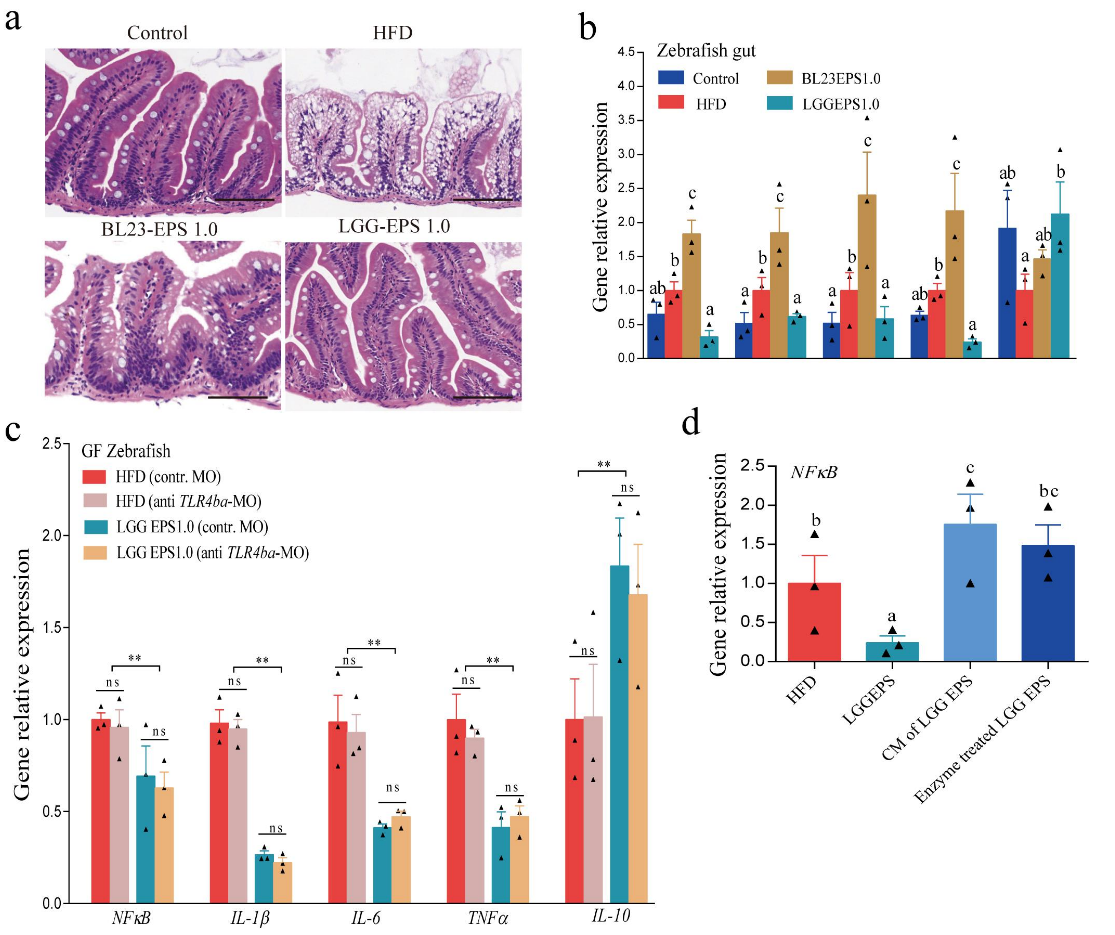

Supplementary Figure 7. BL23 EPS induced intestinal inflammation while LGG EPS showed protective effect. Adult zebrafish (one month old) were fed with the control diet, HF diet, or HF diet supplemented with 1.0% BL23 EPS or 1.0% LGG EPS for four weeks. **(a)** Representative intestines histology images by H&E staining. The scale bar is 50 μm. **(b)** The expression of genes related to inflammation in the intestines as measured by *q*-PCR (n = 3, pool of 3 zebrafish per sample). The expression of genes related in inflammatory **(c)** in GF larvae fed sterile HFD or 1.0% LGG EPS diet and treated with *vivo TLR4ba* morpholino or control morpholino (n = 3, pool of 20 larvae per sample). The expression of *NFκB* **(d)**, in the GF zebrafish fed with HFD, 1.0% LGG EPS diet, HF diet supplemented with 1.0% combination of monosaccharides comprised of LGG EPS or β-Galactosidase treated LGG EPS for one week (n = 3, pool of 20 larvae per sample). Data are expressed as the mean ± SEM. Graph bars labelled with different letters on top represent statistically significant results ($P < 0.05$), whereas bars with the same letter correspond to results that show no statistically significant differences.

Supplementary Table 1. Ingredients and chemical compositions of diets for 1-month-old zebrafish (dry matter, g/kg diet).

Ingredient (g/kg diet)	Control diet	High-fat diet	0.5% EPS 0.5%	1.0% EPS
Casein	400	400	400	400
Gelatin	100	100	100	100
Dextrin	350	250	250	250
Lard oil	0.00	80	80	80
Soybean oil	60	80	80	80
Lysine	3.3	3.3	3.3	3.3
VC phosphate	1.0	1.0	1.0	1.0
Vitamin premix[1]	2.0	2.0	2.0	2.0
Mineral premix[2]	2.0	2.0	2.0	2.0
Monocalcium phosphate	20	20	20	20
Choline chloride	2.0	2.0	2.0	2.0
Sodium alginate	20	20	20	20
Zeolite	39.7	39.7	34.7	29.7
EPS	0.00	0.00	5.0	10.0
Total	1000	1000	1000	1000
Proximate analysis				
Crude protein	42.19	42.19	42.19	42.19
Crude lipid	6.09	15.77	15.77	15.77
Gross energy (KJ/g DM)	18.55	20.85	20.85	20.85

1. Containing the following (g/kg vitamin premix): thiamine, 0.438; riboflavin, 0.632; pyridoxine·HCl, 0.908; *d*-pantothenic acid, 1.724; nicotinic acid, 4.583; biotin, 0.211; folic acid, 0.549; vitamin B-12, 0.001; inositol, 21.053; menadione sodium bisulfite, 0.889; retinyl acetate, 0.677; cholecalciferol, 0.116; *dl*-α-tocopherol-acetate, 12.632.

2. Containing the following (g/kg mineral premix): $CoCl_2\cdot 6H_2O$, 0.074; $CuSO_4\cdot 5H_2O$, 2.5; $FeSO_4\cdot 7H_2O$, 73.2; NaCl, 40.0; $MgSO_4\cdot 7H_2O$, 284.0; $MnSO_4\cdot H_2O$, 6.50; KI, 0.68; Na_2SeO_3, 0.10; $ZnSO_4\cdot 7H_2O$, 131.93; Cellulose, 501.09.

Supplementary Table 2. Ingredients and chemical compositions of diets for larval zebrafish (dry matter, g/kg diet).

Ingredient (g/kg diet)	Control diet	High-fat diet	0.5% EPS	1% EPS
Casein	460	460	460	460
Gelatin	110	110	110	110
Dextrin	180	120	120	120
Lard oil	30	90	90	90
Soybean oil	30	90	90	90
Fish liver oil	20	20	20	20
Soybean lecithin	20	20	20	20
Lysine	1.8	1.8	1.8	1.8
VC phosphate	1.0	1.0	1.0	1.0
Vitamin premix[1]	2.0	2.0	2.0	2.0
Mineral premix[2]	2.0	2.0	2.0	2.0
Monocalcium phosphate	20	20	20	20
Choline chloride	2.0	2.0	2.0	2.0
Sodium alginate	20	20	20	20
Zeolite	101.2	41.2	36.2	31.2
EPS	0.00	0.00	5.0	10
Total	1000	1000	1000	1000
Proximate analysis				
Crude protein	48.09	48.09	48.09	48.09
Crude lipid	9.90	21.66	21.66	21.66
Gross energy (KJ/g DM)	18.60	22.23	22.23	22.23

1. Containing the following (g/kg vitamin premix): thiamine, 0.438; riboflavin, 0.632; pyridoxine·HCl, 0.908; *d*-pantothenic acid, 1.724; nicotinic acid, 4.583; biotin, 0.211; folic acid, 0.549; vitamin B-12, 0.001; inositol, 21.053; menadione sodium bisulfite, 0.889; retinyl acetate, 0.677; cholecalciferol, 0.116; *dl*-α-tocopherol-acetate, 12.632.

2. Containing the following (g/kg mineral premix): $CoCl_2 \cdot 6H_2O$, 0.074; $CuSO_4 \cdot 5H_2O$, 2.5; $FeSO_4 \cdot 7H_2O$, 73.2; NaCl, 40.0; $MgSO_4 \cdot 7H_2O$, 284.0; $MnSO_4 \cdot H_2O$, 6.50; KI, 0.68; Na_2SeO_3, 0.10; $ZnSO_4 \cdot 7H_2O$, 131.93; Cellulose, 501.09.

Supplementary Table 3. Diversity index of gut bacteria of zebrafish fed with control, HFD, BL23 EPS or LGG EPS-supplemented diet for four weeks[1].

Sample	OTUs	Chao1	PD whole tree	Simpson	Shannon
Control	164.3 ± 18.8[b]	157.9 ± 13.6[b]	15.9 ± 1.5[b]	0.67 ± 0.03[b]	2.7 ± 0.3[b]
HFD	220.3 ± 28.2[c]	249.3 ± 25.6[c]	19.6 ± 2.3[c]	0.84 ± 0.03[c]	3.9 ± 0.3[c]
BL23EPS 1.0	140.5±20.8[ab]	137.9±20.5[ab]	14.3±1.5[ab]	0.90±0.01[c]	4.2 ± 0.1[c]
LGGEPS 1.0	93.0 ± 21.6[a]	86.3± 22.7[a]	9.1 ± 2.6[a]	0.50 ± 0.10[a]	1.6 ± 0.4[a]

[1]Values are expressed as the mean ± SEM, n = 3 or 4. Chao1, Chao1 index; OTU, operational taxonomic unit; PD, phylogenetic diversity; Simpson, Simpson's diversity index; Shannon, Shannon diversity index. Means marked with different letters represent statistically significant results ($P < 0.05$), whereas the same letter correspond to results that show no statistically significant differences.

Supplementary Table 4. The predominant gut bacterial phyla in zebrafish fed with control, HFD, BL23 EPS or LGG EPS-supplemented diet for four weeks[1].

Phylum (%)	Control	HFD	BL23EPS 1.0	LGG EPS 1.0
Fusobacteria	42.7 ± 8.6^{c}	28.3 ± 6.6^{b}	8.2±4.6^{a}	44.4 ± 13.8^{c}
Proteobacteria	49.9 ± 10.5^{ab}	43.6 ± 15.5^{a}	79.1±2.9^{b}	53.2 ± 14.4^{ab}
Actinobacteria	4.5 ± 1.8^{a}	11.7 ± 3.9^{b}	6.8±1.7^{ab}	0.7 ± 0.4^{a}
Firmicutes	1.5 ± 0.4^{a}	8.1 ± 3.8^{b}	2.7±0.6^{a}	1.2 ± 0.4^{a}
Bacteroidetes	0.6 ± 0.2^{a}	5.5 ± 3.1^{b}	2.5±1.0^{ab}	0.4 ± 0.2^{a}
Sum (%)	99.0 ± 0.5	97.3 ± 1.8	99.1±0.2	99.9 ± 0.1

[1]Values are expressed as the mean ± SEM, n = 3 or 4. Means marked with different letters represent statistically significant results ($P < 0.05$), whereas the same letter correspond to results that show no statistically significant differences.

Supplementary Table 5. The predominant gut bacterial genera in zebrafish fed with control, HFD, or LGG EPS-supplemented diet for four weeks[1].

Genus (%)	Control	HFD	BL23EPS 1.0	LGG EPS 1.0
Cetobacterium	78.5 ± 3.0[c]	47.6 ± 4.0[b]	11.1±6.2[a]	86.9 ± 4.0[c]
Plesiomonas	1.0 ± 0.5[a]	1.0 ± 0.4[a]	23.3±7.3[b]	0.5 ± 0.3[a]
Aquicella	5.7 ± 1.3[ab]	7.1 ± 3.9[ab]	18.3±7.5[b]	4.3 ± 2.3[a]
Methylocystis	6.2 ± 2.1[ab]	4.8 ± 3.6[ab]	15.5±6.5[b]	0.90 ± 0.4[a]
Lawsonia	0.80 ± 0.3[a]	3.1 ± 1.4[ab]	6.7±1.5[b]	1.7 ± 0.6[a]
Marmoricola	0.43 ± 0.41[a]	6.6 ± 2.1[b]	0.32±0.22[a]	0.07 ± 0.03[a]
Mycobacterium	0.10 ± 0.02[a]	6.1 ± 4.4[b]	0.02±0.01[a]	0.05 ± 0.01[a]
Prevotella	0.57 ± 0.30[a]	4.3 ± 2.4[b]	0.10±0.05[a]	0.17 ± 0.09[a]

[1]Values are expressed as the mean ± SEM, n = 3 or 4. Means marked with different letters represent statistically significant results ($P < 0.05$), whereas the same letter corresponds to results that show no statistically significant differences.

Supporting Information Table 6. Sequences of primers used for *q*-PCR analysis.

Gene Name	Forward (5'→3')	Reverse (5'→3')
β-actin	GGTACCCATCTCCTGCTCCAA	GAGCGTGGCTACTCCTTCACC
(Reference gene)		
rps11	ACAGAAATGCCCCTTCACTG	GCCTCTTCTCAAAACGGTTG
(Reference gene)		
FAS	GGAGCAGGCTGCCTCTGTGC	TTGCGGCCTGTCCCACTCCT
PPARγ	CCTGTCCGGGAAGACCAGCG	GTGCTCGTGGAGCGGCATGT
C/EBPα	AACGGAGCGAGCTTGACTT	AAATCATGCCCATTAGCTGC
PPARα	CTGCGGGACATCTCTCAGTC	ACCGTAAACACCTGACGACG
CPT1	GCATTGACCTTCAGCTCAGC	CTGCCAACACCAGCACGAAC
UCP2	TGCCACCGTGAAGTTTATTG	CCTCGATATTTCACCGGACC
SCD1	TTGCACTGCGTCCCGATGCC	GGCTCGTCGTCGGCAACCTC
ACC1	GCGTGGCCGAACAATGGCAG	GCAGGTCCAGCTTCCCTGCG
DGAT2	CCATACTTGCTGCATATTCC	ATGTCATGATAAACTGCAGC
SREBP-1c	CAGAGGGTGGGCATGCTGGC	ATGTGACGGTGGTGCCGCTG
TNFα	AAGGAGAGTTGCCTTTACCG	ATTGCCCTGGGTCTTATGG
IL-6	TCAACTTCTCCAGCGTGATG	TCTTTCCCTCTTTTCCTCCTG
IL-10	TCACGTCATGAACGAGATCC	CCTCTTGCATTTCACCATATCC
NFkB	GCAAGATGAGAACGGAGACAC	CTACCAGCAATCGCAAACAA
IL-1β	GGCTGTGTGTTTGGGAATCT	TGATAAACCAACCGGGACA
TLR1	CAGAGCGAATGGTGCCACTAT	GTGGCAGAGGCTCCAGAAGA
TLR2	ATACAAGCCAAACGGAAACCT	CTTCTCACATTTCCGCATCAT
TLR3	AAAGGGCTACGTTTGGTGTG	GCATCCTTCAGCGACCCTAA

TLR4ba	TGTCAAGATGCCACATCAGA	TCCACAAGAACAAGCCTTTG
TLR4bb	TGGTGATGAAGAGTCCCTTTCCTA	TCTGCGTGCCAGTAAAAGATCTCA
TLR5a	CATTCTGGTGGTGCTTGTT	CTGCTGCTTCAGGATTGTT
TLR5b	GTGAGGAGCCTGATCCTGATAG	CATACTAAATGTATAATAAGTCTACCATG
TLR18	TTTAGGTCAAGGGGTGGATTAC	CTACTATGTCGGCTGATTGTTCTC
Claudin	TGTTCATCACTGGAGGGCTT	GGAGGATACGAGGGTTTTTC
Claudin-7	CTTGCTCAAAGGGTCAGTCA	GTCCTTTCCAGCTCGTGAAC
Occludin-2	TGGAGATGAGCTTGACACAGATG	CCTTCCTCTAGCCTGTCGAG
Occludin-B	CAAAATCAGGCAAAGGCTTC	AACAATAGTGGCGATGAGCA
Hif-1αa	AGCCGCCACACTTTAGACAT	CCTCTGGATCAAAACCCAAG
Hif-1αb	GCCACACTCTGGACATGAAG	TCAAGAGGTCATCTGGCTCA
Hif-2α	GAGAGCTGTGCAGTCATGGA	GTCGGTTGTCCGTTCTGATT
Arat1a	TCTCCTGGGGGAAAGAAGAT	CCATCGCTGCTTCATCATTA
Arat1b	CTCGCTGAATGCCATAGACA	CCCGAGACGACTGTATTGGT
Noxa	AAGAGCAAACCGCTGTAGTAGA	CATCGCTTCCCCTCCATT
Puma	GAGGAGCAGGCTGTGGAG	GTAGAGGGCATTGATGGTGTC
Mcl1a	AACTCCATCACGCCATACC	TCTGCTCAGCCACCCTCT
Mcl1b	TACCGTCCTCGCCTTCG	TGTCCACAACCCGCCTC
MBL	GTGAGGATGAGAATAAAGTGCT	GTTAGTGAAAGTTAGAGGCTGG
CRP	TCGATAGGGAGGTCATCCTG	GCAGCAGGGTCTTTCTGACT
LBP	GGGACCTATTATGCCCCTCT	AGGCATTTGTAGCTCTGCACT
NOD1	CAGACCAGGTACGCAAAATCTTG	TGAAATACGCCGAGCATTCC
NOD2	GTTATGGTGGGCAAGGACAGA	CATTGGCCTGTCAGCCAGTA
NOD3	CAACATACACACACCGCCTT	TCGTGGCGTAGTTCTTCTTG

NOD4	GTTGGCATTCCTCAGTCGGTTCA	ATCAGTCTTGGTAGTCCATCGGGTT
Muc2.1	AATATGCCTTGCGGAACAAC	GTGCTGAGGTTGCAGAATGA
Lysozyme	GATTTGAGGGATTCTCCATTGG	CCGTAGTCCTTCCCCGTATCA
c3b	CGTCTCCGTACACCATCCATT	GGCGTCTCATCAGGATTTGTTAC
Hepcidin	CACAGCCGTTCCCTTCATAC	AGTATCCGCAGCCTTTATTG
Defbl1	AGGATGCAGCCTCATTCTCTTT	TGAAGCCCCAGAGCATATTTATC
Defbl2	CAATAACACGTCCAATGAAGAGTCT	GGTTTGGGATAGACGACATGAGT
Universal bacteria	CCTACGGGAGGCAGCAG	ATTACCGCGGCTGCTGG
Fusobacterium (phylum)	KGGGCTCAACMCMGTATTGCGT	TCGCGTTAGCTTGGGCGCTG
Proteobacteria (phylum)	TCGTCAGCTCGTGTYGTGA	CGTAAGGGCCATGATG
Cetobacterium (genus)	AGTTTGATCCTGGCTCAGGATG	GAGGCAAGTTCCTTACGCGTT
Plesiomonas (genus)	CTCCGAATACCGTAGAGTGCTATCC	CTCCCCTAGCCCAATAACACCTAAA
