# Supplementary material for: Ability of prebiotic polysaccharides to activate a HIF1α-antimicrobial peptide axis determines liver injury risk in zebrafish
Source: Commun Biol. 2019 Jul 25;2:274. doi: 10.1038/s42003-019-0526-z (PMC6658494; doi:10.1038/s42003-019-0526-z)
Supplement: Supplementary file 5 — Reporting Summary [file 42003_2019_526_MOESM5_ESM.pdf]

nature research

Corresponding author(s): Zhigang Zhou
Last updated by author(s): Jun 5, 2019

Reporting Summary

Nature Research wishes to improve the reproducibility of the work that we publish. This form provides structure for consistency and transparency in reporting. For further information on Nature Research policies, see Authors & Referees and the Editorial Policy Checklist.

Statistics

For all statistical analyses, confirm that the following items are present in the figure legend, table legend, main text, or Methods section.

n/a	Confirmed	
☐	☒	The exact sample size (*n*) for each experimental group/condition, given as a discrete number and unit of measurement
☐	☒	A statement on whether measurements were taken from distinct samples or whether the same sample was measured repeatedly
☐	☒	The statistical test(s) used AND whether they are one- or two-sided<br>*Only common tests should be described solely by name; describe more complex techniques in the Methods section.*
☐	☒	A description of all covariates tested
☐	☒	A description of any assumptions or corrections, such as tests of normality and adjustment for multiple comparisons
☐	☒	A full description of the statistical parameters including central tendency (e.g. means) or other basic estimates (e.g. regression coefficient) AND variation (e.g. standard deviation) or associated estimates of uncertainty (e.g. confidence intervals)
☐	☒	For null hypothesis testing, the test statistic (e.g. *F*, *t*, *r*) with confidence intervals, effect sizes, degrees of freedom and *P* value noted<br>*Give P values as exact values whenever suitable.*
☒	☐	For Bayesian analysis, information on the choice of priors and Markov chain Monte Carlo settings
☒	☐	For hierarchical and complex designs, identification of the appropriate level for tests and full reporting of outcomes
☒	☐	Estimates of effect sizes (e.g. Cohen's *d*, Pearson's *r*), indicating how they were calculated

Our web collection on statistics for biologists contains articles on many of the points above.

Software and code

Policy information about availability of computer code

Data collection	No software was used
Data analysis	GraphPad Prism 6; ImageJ (NIH) v1.42

For manuscripts utilizing custom algorithms or software that are central to the research but not yet described in published literature, software must be made available to editors/reviewers. We strongly encourage code deposition in a community repository (e.g. GitHub). See the Nature Research guidelines for submitting code & software for further information.

Data

Policy information about availability of data

All manuscripts must include a data availability statement. This statement should provide the following information, where applicable:

- Accession codes, unique identifiers, or web links for publicly available datasets
- A list of figures that have associated raw data
- A description of any restrictions on data availability

Microbiota sequencing data in this study are available from the European Nucleotide Archive (ENA) under accession number PRJEB25167. The authors declare that all data supporting the findings of this study are available within the article and its supplementary information files or from the corresponding author (Z. G. Z.) upon reasonable request.

Field-specific reporting

Please select the one below that is the best fit for your research. If you are not sure, read the appropriate sections before making your selection.

☒ Life sciences ☐ Behavioural & social sciences ☐ Ecological, evolutionary & environmental sciences

For a reference copy of the document with all sections, see nature.com/documents/nr-reporting-summary-flat.pdf

Life sciences study design

All studies must disclose on these points even when the disclosure is negative.

Sample size	No statistical calculations were performed to determine sample size.
Data exclusions	No data was excluded from this study.
Replication	For all data presented in the manuscript, we examined at least three independent biological samples to ensure the reproducibility. For each series of the experiments, all attempts at replication were successful.
Randomization	Adult and larval zebrafish were randomly assigned to the experimental groups.
Blinding	Blinding was not performed. However, experiments were independently confirmed and repeated by several of the co-authors in this manuscript. With this strategy, we are confident with all the data presented.

Reporting for specific materials, systems and methods

We require information from authors about some types of materials, experimental systems and methods used in many studies. Here, indicate whether each material, system or method listed is relevant to your study. If you are not sure if a list item applies to your research, read the appropriate section before selecting a response.

Materials & experimental systems

n/a	Involved in the study	
☐	☒	Antibodies
☐	☒	Eukaryotic cell lines
☒	☐	Palaeontology
☐	☒	Animals and other organisms
☒	☐	Human research participants
☒	☐	Clinical data

Methods

n/a	Involved in the study	
☒	☐	ChIP-seq
☒	☐	Flow cytometry
☒	☐	MRI-based neuroimaging

Antibodies

Antibodies used	For immunohistochemistry, C/EBPα (Bioworld, BS1384, 1:200 dilution), DGAT2 (Bioworld, BS60142, 1:300 dilution), PPARα (Bioworld, BS1689, 1:200 dilution), and CPT1 (Bioworld, BS7744, 1:300 dilution).<br>For western blotting, β-actin (CMCTAG, AT0544, 1:1000), HIF-1α (Bioworld, BS3514, 1:1000), TLR4 (Cell Signaling Technology, 14358S, 1:1000).
Validation	These antibodies were validated by the vendor and used according to the vendor's instructions.

Eukaryotic cell lines

Policy information about cell lines

Cell line source(s)	ZFL cells (ATCC number CRL-2643) were obtained from ATCC.
Authentication	ZFL were authenticated by the vendor ATCC.
Mycoplasma contamination	All cell lines were routinely tested for mycoplasma contamination.
Commonly misidentified lines<br>(See ICLAC register)	No commonly misidentified cell lines were used in this study.

Animals and other organisms

Policy information about studies involving animals; ARRIVE guidelines recommended for reporting animal research

Laboratory animals	Danio rerio, AB strain
Wild animals	The study did not involve wild animals
Field-collected samples	The study did not involve samples collected from the field.
Ethics oversight	All experimental and animal care procedures were approved by the Feed Research Institute of the Chinese Academy of Agricultural Sciences Animal Care Committee, under the auspices of the China Council for Animal Care.

Note that full information on the approval of the study protocol must also be provided in the manuscript.
